# Demystifying the Dark Web Opioid Trade: Content Analysis on Anonymous Market Listings and Forum Posts

**DOI:** 10.2196/24486

**Published:** 2021-02-17

**Authors:** Zhengyi Li, Xiangyu Du, Xiaojing Liao, Xiaoqian Jiang, Tiffany Champagne-Langabeer

**Affiliations:** 1 Department of Computer Science Indiana University Bloomington Bloomington, IN United States; 2 The University of Texas Health Science Center at Houston Houston, TX United States

**Keywords:** opioids, black market, anonymous markets and forums, opioid supply chain, text mining, machine learning, opioid crisis, opioid epidemic, drug abuse

## Abstract

**Background:**

Opioid use disorder presents a public health issue afflicting millions across the globe. There is a pressing need to understand the opioid supply chain to gain new insights into the mitigation of opioid use and effectively combat the opioid crisis. The role of anonymous online marketplaces and forums that resemble eBay or Amazon, where anyone can post, browse, and purchase opioid commodities, has become increasingly important in opioid trading. Therefore, a greater understanding of anonymous markets and forums may enable public health officials and other stakeholders to comprehend the scope of the crisis. However, to the best of our knowledge, no large-scale study, which may cross multiple anonymous marketplaces and is cross-sectional, has been conducted to profile the opioid supply chain and unveil characteristics of opioid suppliers, commodities, and transactions.

**Objective:**

We aimed to profile the opioid supply chain in anonymous markets and forums via a large-scale, longitudinal measurement study on anonymous market listings and posts. Toward this, we propose a series of techniques to collect data; identify opioid jargon terms used in the anonymous marketplaces and forums; and profile the opioid commodities, suppliers, and transactions.

**Methods:**

We first conducted a whole-site crawl of anonymous online marketplaces and forums to solicit data. We then developed a suite of opioid domain–specific text mining techniques (eg, opioid jargon detection and opioid trading information retrieval) to recognize information relevant to opioid trading activities (eg, commodities, price, shipping information, and suppliers). Subsequently, we conducted a comprehensive, large-scale, longitudinal study to demystify opioid trading activities in anonymous markets and forums.

**Results:**

A total of 248,359 listings from 10 anonymous online marketplaces and 1,138,961 traces (ie, threads of posts) from 6 underground forums were collected. Among them, we identified 28,106 opioid product listings and 13,508 opioid-related promotional and review forum traces from 5147 unique opioid suppliers’ IDs and 2778 unique opioid buyers’ IDs. Our study characterized opioid suppliers (eg, activeness and cross-market activities), commodities (eg, popular items and their evolution), and transactions (eg, origins and shipping destination) in anonymous marketplaces and forums, which enabled a greater understanding of the underground trading activities involved in international opioid supply and demand.

**Conclusions:**

The results provide insight into opioid trading in the anonymous markets and forums and may prove an effective mitigation data point for illuminating the opioid supply chain.

## Introduction

### Background

Overdoses from opioids, a class of drugs that includes both prescription pain relievers and illegal narcotics, account for more deaths in the United States than traffic deaths or suicides. Overdose deaths involving heroin began increasing in 2000 with a dramatic change in pace, and as of 2014, 61% of drug overdoses involved some type of opioid, inclusive of heroin [1]. Deaths involving fentanyl nearly doubled from the previous year’s rate in 2014, 2015, and 2016 [2]. To reduce opioid-related mortality, there is a pressing need to understand the supply and demand for the product; however, no prior research that provides a greater understanding of the international opioid supply chain has been conducted.

The past 10 years have witnessed a spree of anonymous online marketplaces and forums, mostly catering to drugs in anonymous ways and resembling eBay or Amazon. For instance, SilkRoad, the first modern darknet market and best known as a platform for selling illegal drugs, was launched in February 2011 and subsequently shut down in October 2013 [3]. However, its closure catalyzed the development of multiple other anonymous marketplaces. Compared with traditional opioid supply methods [4], the role of anonymous online marketplaces and forums has become more important because of its stealthiness and anonymity: using this type of virtual exchange, anyone can post and browse the opioid product listings, regardless of their technical background. It raises new challenges for new law enforcement agencies to identify opioid suppliers, buyers, or even takedown the marketplace. Further compounding the issue from a law enforcement perspective, it is nontrivial to obtain complete opioid listings from the darknet markets, interpret the jargon used in the darknet forum, and holistically profile opioid trading and supplying activities.

### Underground Opioid Trading

**Figure 1:**
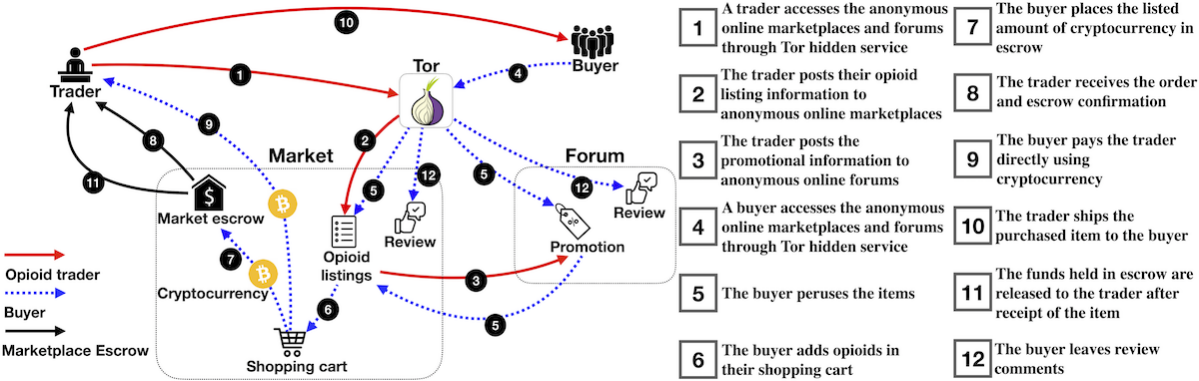
Overview of the opioid trading in the anonymous marketplaces and forums.

Anonymous online marketplaces are usually platforms for sellers and buyers to conduct transactions in a virtual environment. They usually come with anonymous forums for sellers and buyers to share information, promote their products, leave feedback, and share experiences about purchases. To understand how it works, we describe an opioid transaction’s operational steps on the anonymous online marketplaces and forums. We present a view about how such services operate and how different entities interact with each other (Figure 1).

**Figure 2:**
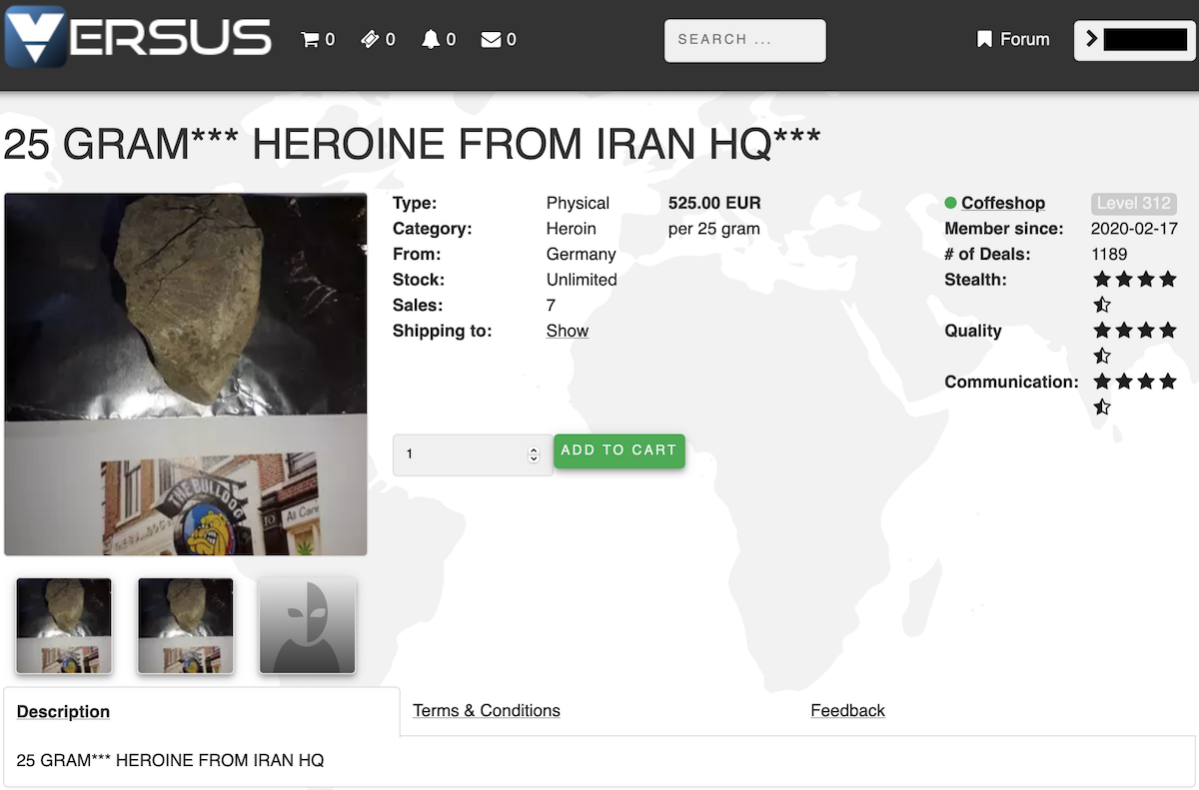
Example of opioid listings in The Versus Project.

**Figure 3:**
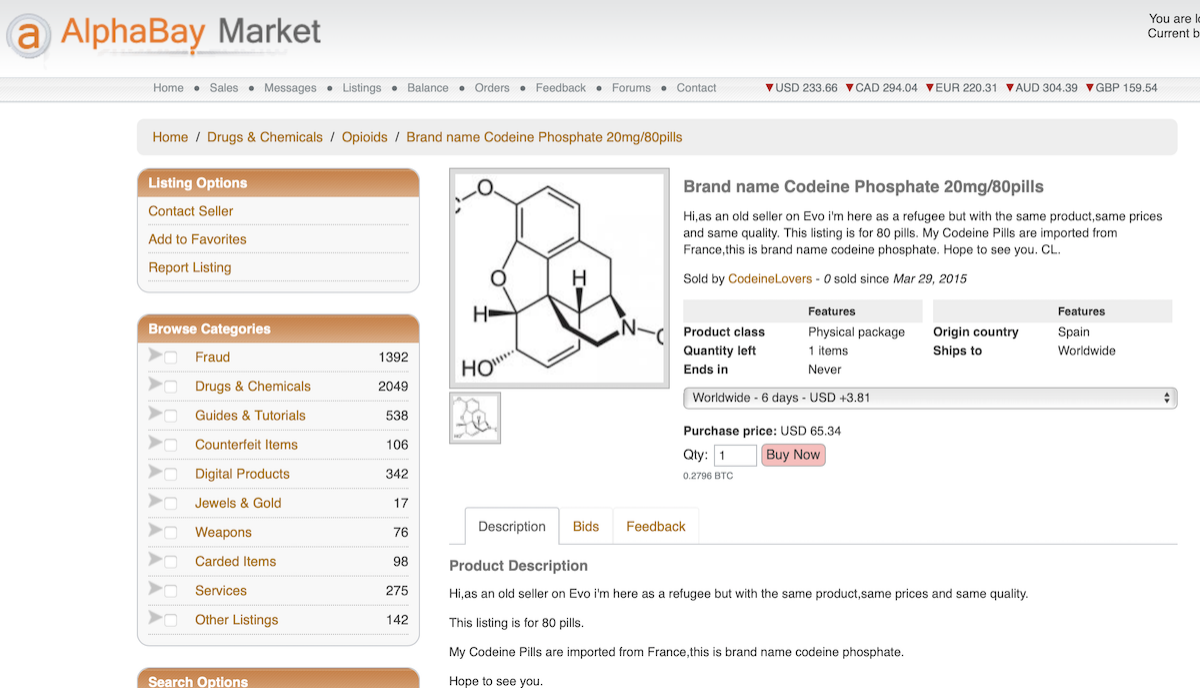
Example of opioid listings in Alphabay.

First, an opioid trader, who intends to list the selling information and find potential customers, will first access the anonymous online marketplaces and forums, using an anonymous browsing tool such as a Tor client or a web-to-Tor proxy (step 1 in Figure 1) [5,6]. Anonymous online marketplaces and forums usually operate as hidden Tor services, which can only be resolved through Tor (an anonymity network). Once connected to the anonymous online marketplaces (eg, The Empire Market and Darkbay), the opioid trader will create an account as a seller and post their opioid listing information (including product, price, origin country, an acceptable shipping destination, payment method, quantities left, shipping options—shipping days or shipping companies, and refund policy; step 2). Figures 2 and 3 illustrate examples of opioid listings in The Versus Project and Alphabay. The opioid trader will also use an anonymous online forum (eg, The Hub Forum) to post promotional information to attract potential customers (step 3).

Suppose that an opioid buyer wants to purchase opioids. The opioid buyer (client) will also access the anonymous online market and create an account in each anonymous marketplace before they can find the listings of opioids (step 4). After perusing the items available on the anonymous online market (step 5), the buyer will add opioids to their shopping cart (step 6). When the buyer wants to check out and make a purchase using cryptocurrency (eg, Bitcoin), if the trader accepts payment through an anonymous online marketplace as an escrow, the buyer will place the listed amount of cryptocurrency in escrow (step 7). Then, the trader receives the order and escrow confirmation (step 8). Otherwise, the buyer will pay the trader directly using cryptocurrency or any other payment method accepted by the trader (step 9) [7]. Note that the escrow mechanism is widely deployed in the anonymous online market because it helps to build trust and resolve disputes between sellers and buyers. When the purchase is made, the opioid trader ships the purchased item to the buyer (step 10). Once the item is received, the buyer finalizes the purchase by notifying the anonymous online marketplace to release the funds held in escrow (step 11) [8,9]. After that, an opioid buyer often leaves review comments under the product listing or discusses the purchasing experience in the forum (step 12).

### Prior Work

Recent years have witnessed the trend of studying opioid use disorders using anonymous marketplaces and forums data [5,8,10] and public social media data (eg, Twitter and Instagram) [11-14]. Gilbert et al [15] described changes in the conceptualizations, techniques, and structures of opioid supply chains and illustrated the diversity of transactions beyond the traditionally linear conceptualizations of cartel-based distribution models. Quintana et al [16] and Fernando et al [17] presented the results of the international drug testing service for opioid commodities from the anonymous marketplaces and showed that most opioid substances contained the advertised ingredient and most samples were of high purity. Dasgupta et al [18] collected opioid listings on Silk Road to analyze the prices of diverted prescription opioids. Duxbury et al [6] evaluated the role of trust in online drug markets by applying exponential random graph modelling to underground marketplace transactions. The results show that vendors’ trustworthiness is a better predictor of vendor selection than product diversity or affordability. Considering social media data (eg, Twitter and Instagram), Nasralah et al [14] proposed a text mining framework to collect opioid data from social media and analyzed the most discussed topics to profile the opioid epidemic and crisis. Mackey et al [13] collected tweets related to the opioid topic to identify illicit online pharmacies and study the illegal sale of opioids in online marketing. Cherian et al [12] gathered codeine misuse data from Instagram posts to understand how misuse is happening and its misused form. Recently, Balsamo et al [11] used a language model to expand vocabularies for opioid substances, routes of administration, and drug tampering on Reddit data from 2014 to 2018 and investigated some important consumption-related aspects of the nonmedical abuse of opioid substances. However, to the best of our knowledge, no large-scale study, which may cross multiple anonymous marketplaces and is cross-sectional, has been conducted to profile the opioid supply chain and unveil characteristics of opioid suppliers, commodities, and transactions.

### Goals

This paper seeks to complement current studies widening the understanding of opioid supply chains in underground marketplaces using comprehensive, large-scale, longitudinal anonymous marketplace and forum data. To this end, we propose a series of techniques to collect data; identify opioid jargon terms used in the anonymous marketplaces and forums; and profile the opioid commodities, suppliers, and transactions. Specifically, we first conducted a whole-site crawl of anonymous online marketplaces and forums to solicit data. We then developed a suite of opioid domain–specific text mining techniques (eg, opioid jargon detection and opioid trading information retrieval) to recognize information relevant to opioid trading activities (eg, commodities, price, shipping information, and suppliers). Subsequently, we conducted a comprehensive, large-scale, longitudinal study to demystify opioid trading activities in anonymous markets and forums.

The contributions of this study are elaborated below. First, we designed and implemented an anonymous marketplace data collection and analysis pipeline to gather and identify opioids data in 16 anonymous marketplaces and forums over a period of almost 9 years between 2011 and 2020. Second, we fine-tuned the semantic comparison model proposed by Yuan et al [19] for opioid jargon detection, which can recognize the opioid jargon as innocent-looking terms and the dedicated terms only used in the anonymous marketplaces and forums. In this way, we generated a rich underground marketplace opioid vocabulary of 311 opioid keywords with 13 categories. Third, we conducted a comprehensive, large-scale, longitudinal study to measure and characterize opioid trading in anonymous online marketplaces and forums. Specifically, using a large-scale and cross-sectional data set, we characterized the activeness and cross-market activities of opioid suppliers, investigated popular opioid commodities as well as their evolution and price trends, and outlined a picture of origins and shipping destinations appearing in opioid transactions in anonymous marketplaces and forums. We believe our findings will provide insight into opioid trading in the anonymous markets and forums for law enforcement, policy makers, and invested health care stakeholders to understand the scope of opioid trading activities and may prove an effective mitigation data point for illuminating the opioid supply chain.

## Methods

### Overview

**Figure 4:**
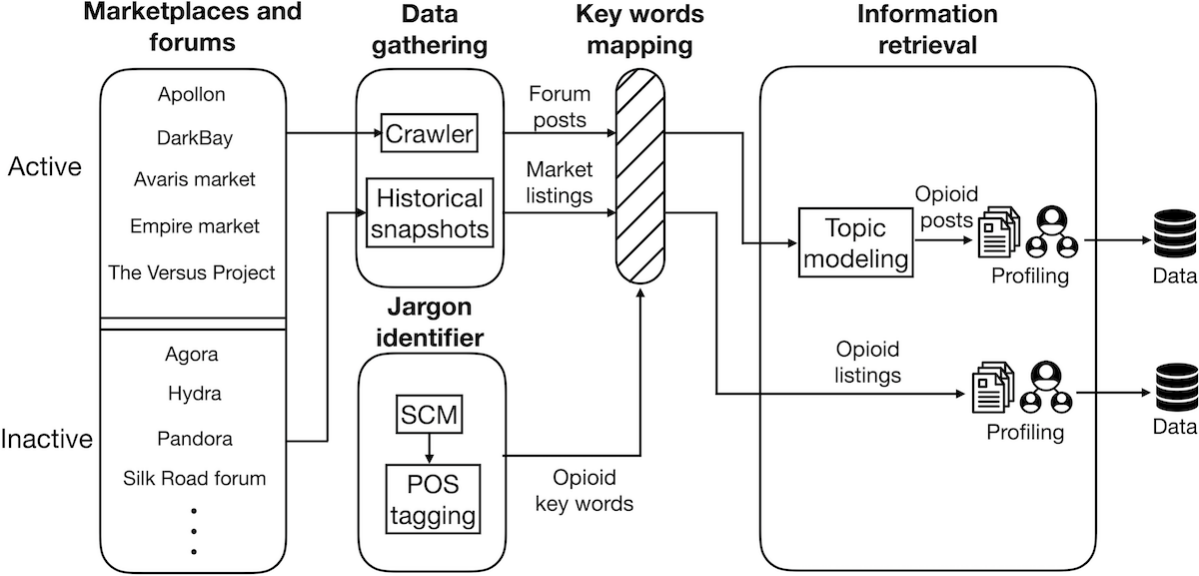
Overview of the methodology workflow. SCM: semantic comparison model, POS: part-of-speech.

This section elaborates on the methodology used to identify opioid trading information in the anonymous market and forums. We illustrate the methodology pipeline (Figure 4). Specifically, we collected approximately 248,359 unique listings and 1,138,961 unique forum traces (ie, threads of posts) from 10 anonymous online marketplaces and 6 forums. We then identified 311 opioid keywords and jargons to recognize 28,106 listings and 13,508 forum traces related to underground opioid trading activities. Finally, we used natural language processing techniques to extract opioid trading information to characterize underground opioid commodities, suppliers, and transactions.

### Data Collection

Our research collected product listings and forum posts from 10 anonymous online market places and 6 forums. Our study determined the underground marketplace and forum list based on darknet site search engines and previous research works [20]. More specifically, we used darknet site search engines (such as Recon, Darknet live, Dark Eye, dark.fail, and DNStats [21,22]) to search underground marketplaces and forums and then manually validated their activeness. In our study, we only selected marketplaces with more than 30 opioid listings. In this way, we gathered 5 active underground marketplaces with opioid listings. Note that some high-profile underground marketplaces and forums are frequently deactivated or have been shut down by law enforcement authorities [23]. Hence, we also gathered snapshots of 5 underground marketplaces and 6 forums collected by the anonymous marketplace archives programs and previous research projects [20].

To collect the listing information of 5 anonymous online marketplaces (ie, Apollon, Avaris, Darkbay, Empire, and The Versus Project), we conducted a whole-site crawl. The crawler was implemented in Python and used the Selenium module to launch browsers and to send crawling requests [24]. To avoid blocking from the marketplace, we provided as an input to the scraper a session cookie that we obtained by manually logging into the marketplace and solving a Completely Automated Public Turing test to tell Computers and Humans Apart (CAPTCHA). However, some sites, namely, the Empire Market, forced users to log out when the life span of the session cookie was expired. In this case, we had to manually repeat the previous process. In addition, we set parameters such as sleeping time to limit the speed of crawling.

In total, we collected 248,359 listings of 10 anonymous online marketplaces between December 2013 and March 2020. For forum corpora, we gathered 1,138,961 traces (spanning from June 2011 to July 2015) from the underground forums The Hub, Silk Road, Black Market, Evolution, Hydra, and Pandora. Table 1 summarizes the data sets used in this study. Note that some forums, such as Pandora and Evolution, were associated with the corresponding marketplaces and mainly served as discussion platforms for marketplace buyers and vendors. In addition, the measurement dates vary across different marketplaces and forums, as they have different life spans.

**Table 1:** Data set summary of marketplaces and forums that were collected for this study.

Name	Type	Lifetime	Measurement dates	Number of traces/listings	Number of opioid traces/listings
Agora	Marketplace	December 2013 to August 2015	January 2014 to July 2015	140,266	12,051
Alphabay	Marketplace	December 2014 to July 2017	December 2014 to July 2015	21,679	1344
Hydra	Marketplace	March 2014 to November 2014	August 2014 to October 2014	3048	218
Pandora	Marketplace	October 2013 to November 2014	December 2013 to November 2014	20,013	1749
Evolution	Marketplace	January 2014 to March 2015	April 2014 to March 2015	54,196	4954
Apollon	Marketplace	May 2018 to March 2020	September 2018 to February 2020	2921	2552
Empire	Marketplace	February 2018 to August 2020	April 2018 to March 2020	2995	2548
The Versus Project	Marketplace	November 2019 to now	November 2019 to March 2020	233	202
Avaris	Marketplace	October 2019 to August 2020	October 2019 to February 2020	291	286
Darkbay	Marketplace	July 2019 to September 2020	July 2019 to February 2020	2717	2112
Black Market	Forum	December 2013 to February 2014	December 2013 to February 2014	52,127	669
Pandora	Forum	October 2013 to September 2014	January 2014 to September 2014	18,640	798
Hydra	Forum	March 2014 to November 2014	April 2014 to September 2014	887	41
The Hub	Forum	January 2014 to now	January 2014 to July 2015	53,973	1082
Evolution	Forum	January 2014 to March 2015	January 2014 to November 2014	166,641	2682
Silk Road	Forum	January 2011 to November 2014	June 2011 to November 2013	846,693	34,519

### Opioid Jargon Identification

Our study used opioid keywords and jargons to recognize listings and forum traces related to underground opioid trading activities. Our opioid jargon identification procedure implemented a modified semantic comparison model [19]. This model employed a neural network–based embedding technique to analyze the semantics of words in different corpora. In particular, in the semantic comparison model, the size of the input layer was doubled while not expanding either the hidden or the output layer. In this way, the same word from 2 different corpora will build separate relations, in terms of weights, from the input to the hidden layer during the training, based on their respective datasets, while ensuring that the contexts of the word in both corpora are combined and jointly contribute to the output of the neural network through the hidden layer. Hence, every word has 2 vectors, each describing the word’s relations with other words in one corpus. In the meantime, these 2 vectors are still comparable because they are used together in the neural network to train a single skip-gram model for predicting the surrounding windows of context words.

Our modification of the semantic comparison model will generate comparable word embeddings for opioid jargon words in legitimate documents (ie, benign corpora embedding) and in underground corpora (ie, underground corpora embedding). Specifically, our modification used a series of opioid keywords collected to generate their benign corpora embeddings and then searched for words whose underground corpora embeddings were close to the opioid keywords’ benign corpora embeddings. We output the top 100 proper nouns in the underground corpora in our implementation, whose embeddings showed the closest cosine distance to the known opioid keywords.

We trained the semantic comparison model using the traces of Reddit as the benign corpora and the traces of the anonymous marketplaces/forums as the underground corpora. The parameters of the model were set as default [19]. Thus, we identified 58 opioid jargon used in the anonymous marketplaces and forums (Table 2). Combining opioid jargon with known opioid product names [25-27], we generated an opioid keyword data set consisting of 311 opioid keywords with 13 categories. We manually validated all keywords and the corresponding categories to guarantee their correctness. Our keywords included almost all the vocabularies of opioid substances mentioned in the study by Balsamo et al [11] and further expanded their results with specific medicine codes, product names, and special colloquialisms (eg, M523, Ultram hydrochloride tramadol 200, and H3 brown sugar).

**Table 2:** Opioid jargons used in the anonymous online marketplaces and forums.

Category	Jargons
Heroin	gunpowder, pearl tar (black pearl tar), speedball, heroin #4, diacetylmorphin, and h3 brown sugar
Fentanyl	chyna (china white), acetylfentanyl (acetyl fentanyl), phenaridine, and duragesic
Buprenorphine	subutex and suboxone
Oxycodone	roxy, roxi, roxies, roxys, oxynorm, A215, K8/K9, M15/30, blueberries, A15, OC30/80, OP80, oxyneo, M523/IP204/C230, bananas, V4812, and CDN 80
Dihydrocodeine	DHC^a^
Oxymorphone	panda and o bomb
Morphine	zomorph, mscontin (ms contin), skenan, oramorph, and kadian
Methadone	amidone, methadose, and chocolate chip cookies
Hydromorphone	hydromorph
Hydrocodone	lortab, norcos, zohydro, IP109/110, and M367
Tramadol	UDT^b^ 200
Codeine	thiocodin and lean
Others	tapentadol, tapalee, and nucynta

^a^Dihydrocodeine bitartrate.

^b^Ultram hydrochloride tramadol.

### Topic Modeling of Forum Posts

Our goal here was to identify anonymous forum posts with the topics of opioid commodity promotion (eg, listing promotion) and review (eg, report fake opioid vendors). We then analyzed these forum posts to profile underground opioid trading behaviors.

To identify forum posts related to opioid commodity promotion and review, our methodology was designed to filter forum posts with opioid keywords and then use a classifier to the posts with the topics of interest. The classifier was built upon transfer learning and a crafted objective function that heavily weighs the penalty of misclassifying a positive instance.

The model training process for opioid promotion and review posts’ detection consists of 3 stages: model initialization, transfer learning, and model refining. First, 2 neural network models with 3 hidden layers are trained on the data sets (Table 3) for model initialization. Then, in the transfer learning stage, the aforementioned models are transferred using manually labeled 800 positive samples (*D_p_*) and 800 negative samples (*D_c_*; Table 3), with the purpose of adjusting the model to fulfill the promotion posts and opioid review detection. Due to the difficulty in collecting the opioid promotion and review posts, the number of positive samples *D_p_* is relatively small compared with negative samples *D_c_*. Note that a sample is only annotated when both of the 2 graduate student annotators agreed with each other. For the data annotation, intercoder reliability measured with Cohen kappa coefficients was 0.74 for promotion post labeling result and was 0.68 for the opioid review labeling result. Considering the imbalance of *D_p_* and *D_c_*, we modified the loss function of the model to make it weigh the penalty of misclassifying a positive instance. The objective function is as follows:

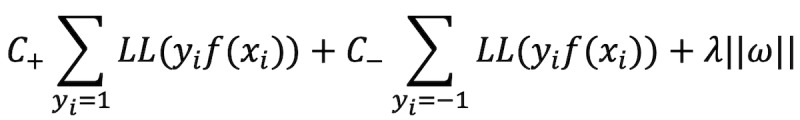



where *LL (z)* is the log loss, that is, 3 log (1 + exp (−z)). *C_+_* and *C_−_* denote the penalty factors for misclassifying the positive and negative instances, respectively, whereas λ is the regularization coefficient and ||ω|| is the regularization term, which is the L1-norm. In the above objective function, *C_+_* is always larger than *C_−_*, which means that the penalty of misclassifying a positive instance is larger than that of a negative instance. In general, the correlation between the penalty factors and the number of samples is set as 
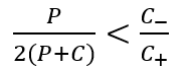
, where *P* and *C* are the sizes of *D_p_* and *D_c_*, respectively.

**Table 3:** Data sets used in the forum post modeling.

Topic	Positive samples (n)			Negative samples (n)	
	Model initialization data set	Annotated anonymous market/forum data set	Model initialization data set		Annotated anonymous market/forum data set
Promotion	Listing descriptions in the marketplace Agora and Alphabay (60,000)	Listing descriptions and product promotions in the anonymous marketplace (1000)	Amazon review data set [28] (30,000) and Yahoo! Answers data sets [29] (30,000)		Nonpromotion (ie, review and question answering) posts in anonymous markets and forums (1000)
Review	Amazon review data set [28] (100,000)	Review posts in an anonymous marketplace (1000)	Yahoo! Answers data sets [29] (100,000)		Nonreview posts in anonymous markets and forums (1000)

Finally, in the model refining stage, the model is trained for other 2 iterations using the same objective function. We manually investigated the results by randomly sampling 10% of data records during each iteration and adding false positive samples into the unlabeled set. Our model was evaluated via 10-fold cross-validation. The review detection model yielded a mean precision of 81.5% and an average recall of 80.1%, whereas for the promotion detection model, it yielded a mean precision of 88.1% and an average recall of 85.1% (Table 4).

**Table 4:** The results and 95% CIs of forum posts’ topic modeling.

Topic modeling methods		Promotion topic		Review topic		
		Precision	Recall		Precision	Recall
**MALLET^a^ document classification, mean (SD)**						
	NaiveBayes	81 (2)	80 (3)		64 (2)	87 (3)
	C45	66 (7)	68 (11)		56 (6)	75 (9)
	Decision tree	83 (3)	51 (3)		71 (2)	61 (4)
**MALLET topic modeling, n (%)**						
	Unsupervised topic modeling	814 (48)	814 (81.40)		939 (53.69)	939 (93.90)
Our model, mean (SD)		88 (1)	85 (2)		82 (1)	80 (1)
Baseline, mean (SD)		84 (1)	84 (3)		76 (3)	74 (2)

^a^MALLET: Machine Learning for Language Toolkit.

We compared our method with the state-of-the-art topic modeling method Machine Learning for Language Toolkit (MALLET) [30] and our model without transfer learning stage (baseline). Our experiment evaluated MALLET on our annotated anonymous marketplace and forum data set (Table 3) using 3 classification algorithms in the document classification tool (package cc.mallet.classify class in MALLET’s JavaDoc API [Application Programming Interface]). In particular, MALLET is retrained and evaluated via 10-fold cross-validation. We also applied the MALLET topic modeling toolkit (package cc.mallet.topics MALLET’s class in JavaDoc API) on the same data set to predict the type of topic. The baseline model was applied directly to the labeled data (Table 3) and evaluated using 10-fold cross-validation. We used the metrics of precision and recall to compare the performance of different topic modeling methods. As shown in Table 4, our results indicate that our approach significantly outperforms MALLET and the baseline model in terms of both precision and average recall.

In this way, we collected 7100 promotion posts and 6408 review posts from forum posts in total.

### Opioid Trading Information Retrieval

For each marketplace listing and forum posts related to opioid promotion, we extracted 8 properties: vendor name, product, price, number of products sold, advertised origins, acceptable shipping destinations, and whether escrow or not. For the forum posts on the topic of the opioid commodity review, we recognized the sentiment of the review. Below, we elaborate on the methodology used to identify each of the properties:

- Vendor name: To identify the vendor name, we designed a parser to identify the authors of the listings and promotional posts by applying platform-specific heuristics, which we manually derived from each marketplace and forum’s HTML templates.
- Product: We recognized the type of opioid in each listing’s description content using the opioid keyword data set generated in the previous step.
- Price: We used a price extraction model [31], which was trained on the underground forum corpora, to extract listing price information (Figures 2 and 3). Our study further determined the per-gram price of opioid products by dividing the listing price by the amount of products. More specifically, we designed a set of regular expressions to extract the amount of opioids sold per listing. For instance, in Figure 3, 1.6 g (20 mg × 80 pills) codeine is sold for US $65.34. Note that following previous works [8], we also dismissed the abnormal price that was greater than 5 times the median of the remaining samples as well as less than 25% of the value of the median.
- Number of products sold: Listings of 5 marketplaces (Alphabay, The Versus Project, Apollon, Empire, and Darkbay) consist of the number of items that have been sold (as shown in Figure 2). Hence, we applied the parser’s feature of identifying the number of sold, which we manually derived from each marketplace’s HTML templates, if such information can be found in the marketplace.
- Advertised origins and acceptable shipping destinations: We parsed the advertised origins and acceptable shipping destinations from the HTML template of marketplace listings and used the country name dictionary to find the country names from a forum post. We considered the contextual information based on the keywords *ship*, *origin*, and *destination*.
- Whether escrow: In the marketplaces Alphabay, Apollon, and Empire, the product listing usually has a field to indicate whether the escrow is supported. Hence, we designed a parser to obtain this information. In forum posts, we used the keyword *escrow* to match each forum post with the topic of promotion to find out whether the trader accepts *escrow*.
- Review sentiment: To investigate the sentiment of the opioid product reviews, we applied the chi-square score–based sentiment analysis model to classify the product review into positive and negative [32].

To evaluate the aforementioned methods for extracting properties, we randomly chose 1000 listings for each property and manually annotated the properties as ground truth. We evaluated our method on our annotated data set, which yields an accuracy of over 90% for each property extraction, as shown in Table 5.

**Table 5:** The results of calculating accuracy of opioid information retrieval.

Property	Number of ground truth, n	Accuracy, n (%)
Vendor name	1000	1000 (100)
Product	1000	954 (95.40)
Price	1000	1000 (100)
Number of products sold	1000	1000 (100)
Advertised origins	1000	1000 (100)
Acceptable shipping destinations	1000	1000 (100)
Whether escrow	1000	1000 (100)
Review sentiment	1000	926 (92.60)

## Results

### Landscape

In total, we collected 248,359 listings from 10 anonymous online marketplaces and 1,138,961 traces (ie, threads of posts) from 6 underground forums. Among them, we identified 28,106 opioid product listings and 13,508 opioid-related promotional and review forum traces from 5147 unique opioid suppliers’ IDs and 2778 unique opioid buyers’ IDs. As observed in our data set, the top 3 marketplaces with the most opioid listings are Agora, Evolution, and Apollon.

In our study, we found that 23.78% (9896/41,614) listings and traces were identified with the help of 58 opioid jargons (Table 2). Among them, *suboxone* and *subutex* medicines are most frequently mentioned by 2917 times in listings and traces in 10 platforms, followed by roxy series (ie, roxy, roxi, roxies, and roxys) with 2022 times and *Lean* with 1256 times. Both *K9* and *M30* were mostly found in Darkbay, within 384 listings in the year 2020, whereas *Lean* appeared 141 times in Empire listings.

Note that we should not overestimate the number of suppliers and buyers given the number of IDs found in this research, but we regarded it as the upper-bounded number of the opioid suppliers and buyers. This is because the same user could have different IDs, and the same ID in different marketplaces can point to different users. Owing to the anonymity of the underground marketplaces and forums, there exists no ground truth to link users with their IDs.

### Characteristics of Commodities

We list the top 5 opioids with most listings and their average prices in 2014, 2015, 2019, and 2020 (Table 6). In general, heroin was found to be the most popular item on the anonymous online market, followed by oxycodone. We also noticed that heroin dropped by 60%, whereas codeine increased by 32% from 2014 to 2019, which is roughly in line with the temporal trend of popularity of opioid substances on Reddit from 2014 to 2018, as mentioned in the study by Balsamo et al [11]. More remarkably, we observed 2011 listings of *China white* (or the slang term *chyna*), a *designer opioid* with significant medical concerns due to its deadly clinical manifestations, in 6 marketplaces and 5 forums. The earliest listing was observed on the SilkRoad in June 2011. Moreover, we notice that most of the top opioids have a lower mean price than their retail prices. For instance, the United Nations Office on Drugs and Crime [33] reported that the average retail price of heroin was US $267 per gram in the United States in 2014 and 2015, which is almost twice as much as the price in anonymous marketplaces (US $130-190 per gram). In addition, we see a trend of drop in price from 2014 to 2019 of some opioids such as heroin, oxycodone, and fentanyl, which may lead to severe overdoses.

**Table 6:** Popular opioids according to different years. Note that data for 2020 only included data from January to March; price per gram is in US $.

2014			2015			2019			2020			
Name	Number of listings	Price (per gram), mean (SD)	Name	Number of listings	Price (per gram), mean (SD)	Name	Number of listings	Price (per gram), mean (SD)		Name	Number of listings	Price (per gram), mean (SD)
Heroin	4251	129.5 (99.9)	Heroin	2408	185.6 (138.4)	Heroin	1697	73.0 (49.8)		Heroin	611	67.0 (37.8)
Oxycodone	3086	660.3 (445.3)	Oxycodone	2079	1239.1 (843.0)	Oxycodone	1078	520.8 (444.9)		Oxycodone	356	590.6 (450.4)
Fentanyl	1397	1116.4 (647.5)	Fentanyl	1450	1546 (909.4)	Codeine	418	80.3 (61.1)		Fentanyl	149	154.2 (123.2)
Buprenorphine	934	2764.9 (2007.8)	Buprenorphine	571	4243.7 (3006.1)	Tramadol	331	16.1 (11.7)		Buprenorphine	90	2083.7 (1471.7)
Tramadol	839	21.5 (14.4)	Tramadol	555	29.8 (29.1)	Fentanyl	282	247.8 (172.1)		Hydrocodone	89	1183.1 (374.0)

**Figure 5:**
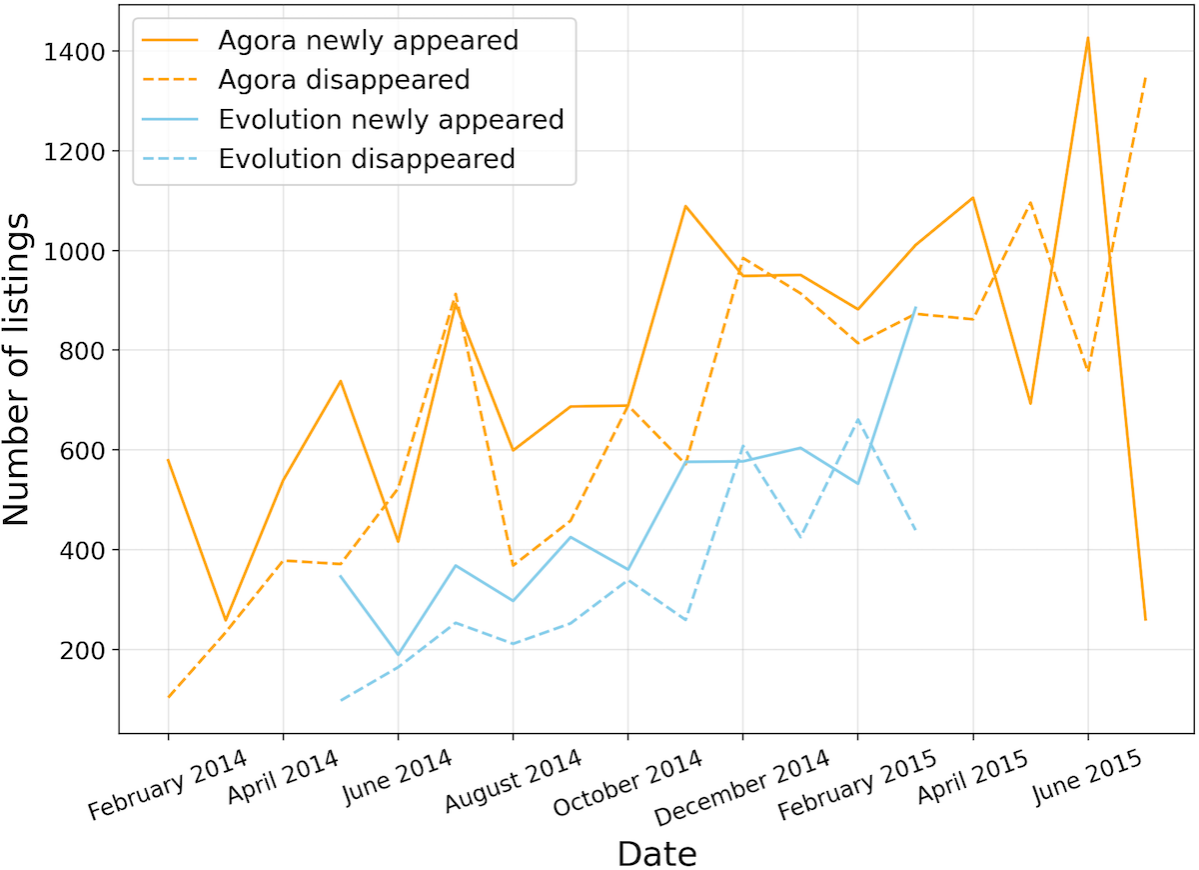
Number of monthly newly-appeared and disappeared listings in the marketplaces Agora and Evolution.

When evaluating the activeness of the underground opioid listing, we measured the monthly *newly appeared* and *disappeared* listings in 2 anonymous online marketplaces: Agora and Evolution. Figure 5 shows the results. We observed that large amounts of opioid listings were newly posted every month in the Agora marketplace, which had a relatively higher rate of increase than the disappeared rate in terms of listings. A similar scenario was observed in the marketplace Evolution. We also observed an increase in newly appeared listings in the Agora marketplace in April 2015. This may be because of the shutdown of the Evolution marketplace in March 2015.

We observed the same listings posted in different marketplaces and illustrate the dependency of the same opioid listings among different marketplaces. Note that we determined if 2 listings are identical by matching the same elements (ie, listing’s title and description information and the vendor’s name) in 2 listings. We observed that the marketplaces of Agora and Evolution shared 530 opioid listings from 290 unique supplier IDs. The opioid commodity with most listings across different marketplaces was *#4 White Vietnamese Heroin*, which can be found in the marketplaces Agora, Evolution, Hydra, and Pandora.

### Characteristics of Suppliers

To understand the scale of opioid suppliers on the anonymous online market, we scanned the listings of 10 marketplaces and the promotional posts of 6 forums to extract the account information from 5147 unique opioid suppliers. By the time Agora was shut down in August 2015, 916 opioid suppliers were found, with an average number of listings of 13 per supplier. We observed that the opioid suppliers with most listings is *mikesales,* which contributes to 817 opioid listings for the marketplace Darkbay, whereas the opioid supplier ID which was observed in most marketplaces is *DeepMeds,* which posted the similar listings of buprenorphine, codeine, and narcotic in 6 different marketplaces from 2014 to 2020. The average number of listings that a legit supplier posts on Amazon is approximately 37 [34,35], which is far less than those posted by suppliers in anonymous marketplaces. It is possible that those suppliers in darknet marketplaces are hidden under an anonymous environment with little to no limitations.

**Figure 6:**
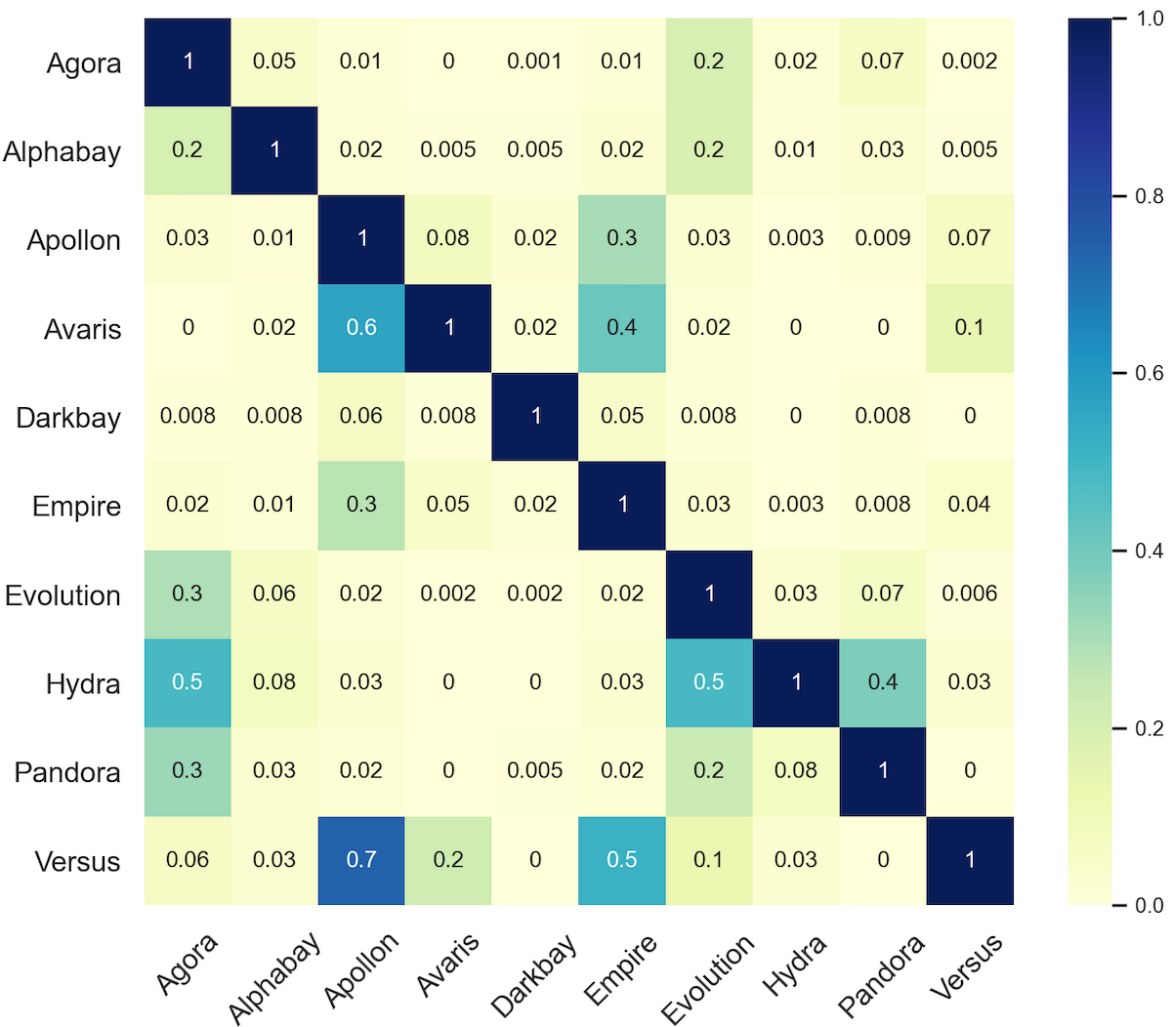
Co-occurrence of the same opioid suppliers across different anonymous marketplaces.

To better understand the potential bundling relationship of opioid suppliers across different marketplaces, we calculated the Jaccard similarity coefficient between the suppliers in different marketplaces (Figure 6). We found that 182 opioid supplier IDs appeared in both the marketplaces Evolution and Agora from January 2014 to July 2015. In particular, we observed that 84 opioid supplier IDs synchronized similar product listings in both marketplaces at the same time. This might be because the suppliers tend to promote their products across various marketplaces and to increase sales.

Inspired by the work [36] investigating the supplier migration phenomenon between underground marketplaces, we also evaluated the migrant suppliers who, for the first time, began to trade in a new market, *m’,* after the closure of marketplace *m*. To this end, we first collected the marketplace’s lifespan using the Gwern archive [37], as shown in Table 1, and then compared the supplier lists in each marketplace to investigate supplier migration. We found that 28 and 35 suppliers in Evolution moved to Agora and Alphabay, respectively, after March 2015 when Evolution was shut down, which is aligned with the finding of users’ migration between these 3 markets by El Bahrawy et al [36], who used a bitcoin address instead of supplier IDs for user matching. In addition, we observed that 10 and 9 suppliers in Apollon and Empire, respectively, migrated from Agora 3 years after it shut down. Some of those suppliers’ IDs (ie, *A1CRACK* and *DiazNL*) are neither common words nor have special meaning. We hypothesized that these IDs might be linked to the same supplier. Those suppliers kept using the same IDs for years to gain reputation and familiarity from buyers.

In addition, 204 suppliers were reported as scammers in the anonymous forums of the Evolution, SilkRoad, Pandora, and The Hub. It is not surprising to find that the top 3 marketplaces and forums that found the most scam reports were Evolution, Silk Road, and Pandora, as the scam reports mostly come from the associated forums of the marketplaces [38].

### Characteristics of the Drug Transaction

When inspecting the advertised origins and the acceptable shipping destinations on the opioid listings from 7 marketplaces (Apollon, Avaris, Alphabay, Hydra, Pandora, Empire, and Versus), we observed that most of the opioid commodities were shipped from the United States, followed by the United Kingdom, Germany, Netherlands, and Canada (Table 7). This finding is roughly in line with the observation of 57 opioid vendors’ origin in a marketplace named Cryptomarket during the period of October 2015 through April 2016, which was reported by Duxbury et al [6]. In addition, we did not observe big changes in the top opioid commodity origins from 2014 to 2020. Particularly, as shown in Table 8, the United States and the United Kingdom are always in the top 5 advertised origins among years.

**Table 7:** The advertised origin countries.

Name of country	Percentage of origin countries in opioid listings, n (%)
United States	3520 (35.3)
United Kingdom	1648 (17.1)
Germany	1459 (14.7)
Netherlands	897 (9)
Canada	622 (6.2)
France	479 (4.8)
Australia	398 (4)
India	200 (2)
Spain	160 (1.6)
Sweden	80 (0.8)
Japan	73 (0.7)
Italy	60 (0.6)
Singapore	55 (0.6)
Belgium	51 (0.5)
Switzerland	50 (0.5)
Portugal	22 (0.2)
Afghanistan	18 (0.2)
Denmark	17 (0.2)
Czech Republic	14 (0.1)
China	11 (0.1)

**Table 8:** Top 5 advertised origin countries according to different years. Note that data for 2020 only included data from January to March.

2014		2015		2019		2020	
Country	Number of appearance in listings	Country	Number of appearance in listings	Country	Number of appearance in listings	Country	Number of appearance in listings
United States	778	United States	603	United States	1394	United States	680
Germany	646	France	188	United Kingdom	1269	Netherlands	250
Netherlands	145	Canada	151	Germany	537	United Kingdom	185
United Kingdom	142	Australia	112	Netherlands	451	Germany	176
Canada	131	United Kingdom	96	Canada	287	Australia	75

Considering the shipping destination, we observed that the majority of opioid commodities were shipped worldwide 36.37% (5654/15,546), followed by shipping to the United States only 19.35% (3008/15,546), Europe only 10.52% (1635/15,546), and the United Kingdom only 5.46% (849/15,546).

To understand customer satisfaction, we conducted a sentiment analysis on 624 review posts related to 190 opioid suppliers from 4 marketplaces: Agora, Alphabay, Pandora, and Evolution. We observed that 145 opioid suppliers had 378 positive reviews, whereas 102 opioid suppliers had at least one negative review. For instance, the opioid supplier from the SilkRoad with the user ID *c63amg* received a satisfaction rating of 76%, even though they had the most negative reviews (n=11). We notice that their negative reviews mostly came from one buyer in late 2012 and early 2013, who complained “his Heroin is getting from order to order worse.”

As observed in our data set, the opioid suppliers in the marketplaces Evolution, Pandora, and Silk Road accepted escrow as a method of payment. However, most of the suppliers only used escrow for small orders. This shows the weak platform trust of opioid suppliers. In fact, the shutdown of the marketplace Evolution was discovered to be an exit scam, with the site’s operators shutting down abruptly to steal the approximately US $12 million in bitcoins that it was holding as an escrow [39].

## Discussion

### Principal Findings

Our study identified 41,000 opioid trade–related marketplace listings and forum posts by analyzing more than 1 million listings and posts in multiple anonymous marketplaces and forums, which is the largest underground opioid trading data set ever reported. We found evidence through extensive analyses of the anonymous online market of pervasive supply, which fuels the international opioid epidemic. Nontraditional methods, as presented here by studying the online supply chain, present a novel approach for governmental and other large-scale solutions. When interpreted by professionals, our initial results demonstrate useful findings and may be used downstream by law enforcement and public policy makers for impactful structural interventions to the opioid crisis. Although a large body of current research is focused on pathways for treatment of opioid use disorder and analyzing deaths per treatment capacity of substance use providers, these research areas are limited to the demand side of the opioid epidemic [40,41]. We believe that the findings and pattern analyses presented here, which place concentration on the supply side, might suggest a new direction to focus and will serve as a useful complement to current research conducted within the domain of addiction medicine.

### Limitations

We acknowledge some limitations of our study. For example, there might be varying types of heroin or fentanyl, but we could not subcategorize them due to the lack of precise ontology. Addressing this challenge requires deep domain knowledge and expertise, which is constantly evolving. Another limitation is pointed out in the paper that multiple online suppliers might belong to the same vendor. This problem might be addressed by studying the product overlapping patterns over time to merge suppliers, which might reveal more interesting hierarchical clustering patterns. Another important source of information is the trading cash flow, which is recorded in the block chain and might contribute to a comprehensive view of the supply-demand relationship. We did not include such analyses due to the time and scope constraints, and it is a topic that we plan to investigate further.

### Conclusions

In our study, a total of 248,359 listings from 10 anonymous online marketplaces and 1,138,961 traces (ie, threads of posts) from 6 underground forums were collected. Among them, we identified 28,106 opioid product listings and 13,508 opioid-related promotional and review forum traces from 5147 unique opioid suppliers’ IDs and 2778 unique opioid buyers’ IDs. Our study characterized opioid suppliers (eg, activeness and cross-market activities), commodities (eg, popular items and their evolution), and transactions (eg, origins and shipping destination) in anonymous marketplaces and forums, which enabled a greater understanding of the underground trading activities involved in international opioid supply and demand.

To the best of our knowledge, a comprehensive overview of the opioid supply chain in the anonymous online marketplaces and forums, as well as a measurement study of trading activities, is still an open research challenge. This is the first study to measure and characterize opioid trading in anonymous online marketplaces and forums. From our measurement, we concluded that anonymous online marketplaces and forums provided easy-access platforms for global opioid supply. These findings characterizing mass opioid suppliers, commodities, and transactions on anonymous marketplaces and forums can enable law enforcement, policy makers, and invested health care stakeholders to better understand the scope of opioid trading activities and provide insight into this new type of opioid supply chain.

## Abbreviations

API: Application Programming Interface
MALLET: Machine Learning for Language Toolkit
